# Impact of maternal *Bifidobacterium bre*ve M-16V and scGOS/lcFOS supplementation during pregnancy and lactation on the maternal immune system and milk composition

**DOI:** 10.3389/fimmu.2024.1418594

**Published:** 2024-06-21

**Authors:** Laura Sáez-Fuertes, Garyfallia Kapravelou, Blanca Grases-Pintó, Malen Massot-Cladera, Manuel Bernabeu, Karen Knipping, Johan Garssen, Raphaëlle Bourdet-Sicard, Margarida Castell, Maria José Rodríguez-Lagunas, Maria Carmen Collado, Francisco José Pérez-Cano

**Affiliations:** ^1^ Physiology Section, Department of Biochemistry and Physiology, Faculty of Pharmacy and Food Science, University of Barcelona (UB), Barcelona, Spain; ^2^ Nutrition and Food Safety Research Institute (INSA-UB), Santa Coloma de Gramenet, Spain; ^3^ Institute of Agrochemisty and Food Technology (IATA-CSIC), National Research Council, Valencia, Spain; ^4^ Division Immunology, Danone Nutricia Research, Utrecht, Netherlands; ^5^ Division Pharmacology, Institute for Pharmaceutical Sciences, Utrecht University, Utrecht, Netherlands; ^6^ Life Science and Digital Health, Danone Global Research & Innovation Center, Gif-sur-Yvette, France; ^7^ Biomedical Research Centre in Physiopathology of Obesity and Nutrition (CIBEROBN), Institute of Salud Carlos III, Madrid, Spain

**Keywords:** *Bifidobacterium breve* M-16V, short chain galacto-oligosaccharides (scGOS), long chain fructo-oligosachairdes (lcFOS), pregnancy, breastfeeding

## Abstract

**Introduction:**

Maternal synbiotic supplementation during pregnancy and lactation can significantly influence the immune system. Prebiotics and probiotics have a positive impact on the immune system by preventing or ameliorating among others intestinal disorders. This study focused on the immunomodulatory effects of *B. breve* M-16V and short chain galacto-oligosaccharides (scGOS)/long chain fructo-oligosachairdes (lcFOS), including systemic and mucosal compartments and milk composition.

**Methods:**

Lewis rats were orally administered with the synbiotic or vehicle during pregnancy (21 days) and lactation (21 days). At the weaning day, small intestine (SI), mammary gland (MG), adipose tissue, milk, mesenteric lymph nodes (MLN), salivary gland (SG), feces and cecal content were collected from the mothers.

**Results:**

The immunoglobulinome profile showed increased IgG2c in plasma and milk, as well as elevated sIgA in feces at weaning. The supplementation improved lipid metabolism through enhanced brown adipose tissue activity and reinforced the intestinal barrier by increasing the expression of *Muc3*, *Cldn4*, and *Ocln*. The higher production of short chain fatty acids in the cecum and increased *Bifidobacterium* counts suggest a potential positive impact on the gastrointestinal tract.

**Discussion:**

These findings indicate that maternal synbiotic supplementation during gestation and lactation improves their immunological status and improved milk composition.

## 1 Introduction

Pregnancy and lactation are crucial for a correct development of the infant. These two periods are highly influenced by external stimuli including environmental, nutritional, and lifestyle factors. Nutrition, including the nourishment status and pattern of food intake, impacts on physiological and metabolic responses (1, 2). During gestation the placenta serves as a bridge for nutrients, hormones, cytokines (CK), immunoglobulins (Igs) and other bioactive molecules. In humans, IgG crosses the placenta through an active mechanism due to the presence of the neonatal fragment crystallizable receptor (FcRn) expressed by the cells of the syncytiotrophoblast (3). During lactation breast milk (BM) works as a transference vehicle for nutrients and bioactive compounds including Ig and CK. Its composition is dynamic and changes to supply the nutritional requirements of the infant (4). Thus, during these periods the maternal nutrition influences the composition of the BM (5).

The use of food complements such as probiotics, prebiotics, synbiotics and postbiotics has been growing during the last few years (6) for different purposes such as their anti-infective action. Probiotics reduce the colonization of pathogens, prebiotics promote the growth of the beneficial microbes of the intestine and, synbiotics act as a combination of both taking the advantages of probiotics and prebiotics (6). In 2020, synbiotics were defined as “a mixture comprising live microorganisms and substrate(s) selectively utilized by host microorganisms that confers a health benefit on the host” (7). Clinical and preclinical studies have verified that the supplementation with these microbial modulators improves the gastrointestinal health and infection resolution (8, 9). In some studies, these effects have been associated with the microbiota and its impact on the immune system (6, 9).

Until now, only a few studies have reported beneficial effects of probiotics and prebiotics supplementation during gestation and lactation on the newborn (10). However, less information is known about the impact of synbiotic supplementation on the maternal immunity. Hence, this study aims to evaluate the impact of a synbiotic mixture, particularly composed of *Bifidobacterium breve* M-16V (10^9^ CFU) and short-chain galacto-oligosaccharide (scGOS) and long-chain fructo-oligosaccharide (lcFOS) at ratio 9:1 during gestation and lactation on the maternal immune system. These probiotic and prebiotic have been linked to immunomodulatory effects in previous studies (11–16).

## 2 Materials and methods

### 2.1 Animals

Seven-week-old Lewis rats (16 females and 8 males) were obtained from Janvier Labs (La Plaine Saint Denis Cedex, France). After one week of acclimatization, females were randomly distributed into two groups: Reference (REF, n=8) or Synbiotic (SYN, n=8). At the same day, females were introduced into the male cages for one week, and then separated into individual cages. Since the mating day, the female rats of the SYN group were supplemented daily during gestation (21 days) and lactation (21 days) with the synbiotic mixture, and the REF group received a matched volume of saline solution. Animals were fed with a commercial diet corresponding to the American Institute of Nutrition 93G formulation (17) and water *ad libitum.* Rats were allowed to deliver naturally, and the day of birth was considered as day 1 of pups. Pups had free access to the nipples and rat diet during the entire study. The experiment was finally executed with the 5 dams of the SYN group and 6 dams of the REF group that became pregnant.

Animal room conditions (temperature and humidity) were controlled in a 12 h light – 12 h dark cycle in a negative pressure chamber at Animal Facility of the Diagonal Campus, Faculty of Pharmacy and Food Science, from the University of Barcelona. All the experimental procedures were previously approved by the Ethics Committee for Animal Experimentation (CEEA) of the University of Barcelona (UB) (Ref 240/19) and from the Catalan Government (Ref.10933).

### 2.2 Synbiotic supplementation

Daily administration of the synbiotic or vehicle was performed during the gestation and lactation periods always at the same time range of the day. The synbiotic solution was obtained by mixing *Bifidobacterium breve* M-16V (10^9^ CFU) with scGOS/lcFOS 9:1 concentration. *Bifidobacterium breve* M-16 V has been purchased from Morinaga Milk Industry, Tokyo, Japan. GOS/FOS is a mixture of GOS (Vivinal GOS, Borculo Domo, Zwolle, The Netherlands) with a degree of polymerization (dp) of 3–8, as well as long-chain FOS (Raftiline HP, Orafti, Wijchen, The Netherlands; average dp > 23) in a 9:1 ratio. The products had a purity of 47.6% for GOS and 94.5% for FOS. The dose of scGOS/lcFOS was approximately 2% of an established daily food intake of 40 g. The mix was extemporaneously prepared by the mixture of the prebiotic and the probiotic dissolved in physiological saline solution. One mL of the synbiotic (10^9^ CFU/rat/day) or saline solution was intragastrically administered through an oral gavage during pregnancy. After birth, dams were separated from the pups for animal handling and the volume administered was increased to 1.5 mL. All supplements were kindly provided by Danone Nutricia Research (Utrecht, The Netherlands). The control group received the same volume of saline with same amount of corn starch as the treated group.

### 2.3 Sample collection and processing

Animal body weights (BW), and food and water consumption were monitored daily, and feces were collected weekly during the study. The relative humidity and the pH of the feces was monitored in fresh after collection. At the weaning day, dams were isolated from the pups 1 h before milk extraction to allow the milk to accumulate in the mammary gland (MG). Then, dams were anesthetized with 10 mg/100 g of ketamine (Merial Laboratories S.A., Lyon, France) and administered intraperitoneally with 2 Ul of oxytocin (Syntocinon 10 U.I./mL, Alfasigma S.L., Bologna, Italia)thirty min after administering oxytocin, the milking process began by gently and manually stimulating the teat from its base to the top. The milk was collected with an automatic pipette in sterilized tubes, centrifuged (12000 *g*, 5 min, 4°C) and then the lactic serum (LS) was obtained and stored at -80°C.

Finally, dams were re-anesthetized with ketamine (90 mg/kg) and xylazine (10 mg/kg; Bayer A.G., Leverkusen, Germany). First, blood was obtained by cardiac puncture and collected in heparin tubes for the hematologic analyses. Then, samples were centrifuged (1000 *g*,10 min 4°C). Also, intestinal and adipose tissues, intestinal and cecal contents, salivary glands (SG), mesenteric lymph nodes (MLN), MGand spleen were collected and immediately processed or stored at -20°C or -80°C for future analysis.

BW was monitored daily and at the end point body and tail lengths were measured to calculate the body mass index (BMI) (*weight/length* ^2^ (g/cm^2^)) and the Lee Index (*weight* ^0.33^ */length)* × *1,000* (g^0.33^/cm). The weight of different organs was recorded including thymus, spleen, liver, heart, kidney, large intestine, and small intestine (SI) (length and wide were also measured).

### 2.4 Isolation of mesenteric lymph nodes and spleen lymphocytes

MLN and spleen cells were isolated as previously described (18). For splenic cells, an additional step was required to eliminate erythrocytes by an osmotic lysis. Conditions were immediately restored by adding PBS (Phosphate-buffered saline) to avoid lymphocytes death (19). Cell viability and concentration was analyzed by Countess™ Automated Cell Counter (Invitrogen™, ThermoFisher Scientific, Barcelona, Spain) based on Trypan Blue staining.

### 2.5 Small intestine sampling

SI was processed for diverse analysis. Two portions of 1 cm from the middle part of the intestine were collected for histomorphometry and gene expression analysis. For gene expression analysis the intestine portion was immersed in RNAlater (Ambion, Life technologies, Madrid, Spain), kept at 4°C for 24 h and then stored at -20°C. The remaining proximal part of the SI was opened lengthwise and cut in 0.5 cm pieces and incubated with PBS in a shaker (37°C for 10 min) to recover the gut wash (GW). The content of the distal part of the SI (intestinal content, IC) was collected for microbiota analysis.

### 2.6 Histology

The SI and adipose tissues (white adipose tissue (WAT) and brown adipose tissue (BAT)) were fixed in 4% buffered formaldehyde for 24 h at room temperature. Then, samples were rinsed in PBS solution for 3 h until dehydrated in graded ethanols (70%, 90% and 100%) and permeated in xylene (Panreac Química SLU, Barcelona, Spain). Afterwards, samples were embedded in melted paraffin (Merck, Madrid, Spain). Paraffin sections (5 µm) were stained using hematoxylin-eosin (HE). Observation of the samples was performed under the microscope (Olympus BX41 and Camera Olympus XC50, Olympus Barcelona, Spain). Representative photos were made for each sample of WAT (20x), BAT (40x) and intestine (10x) and were analyzed using Image J (Image Processing and Analysis in Java, National Institute of Mental Health, Bethesda, MD, USA). In SI, the length and width of microvilli were measured. In WAT, adipocyte area as well as the number of adipocytes per section were quantified. In BAT, the number of nuclei, the area of LDs as well as the number of LDs with a size greater than 50 µm^2^ were measured.

### 2.7 Immunoglobulin quantification

Different tissues and samples were processed for Ig quantification by Enzyme-Linked ImmunoSorbent Assay (ELISA) (Bethyl, Laboratories Inc., Montgomery, TX, USA) and/or ProcartaPlex™ Multiplex immunoassay (eBioscience, San Diego, CA, USA).

Secretory (s)IgA quantification was performed by a sandwich ELISA technique in milk, salivary and mammary glands, MLN, cecal and fecal homogenates. Additionally, sIgA and IgM were evaluated in GW. Both Igs were quantified following the previous described protocol (20), and absorbance results were measured with a microplate photometer (Labsystems Multiskan, Helsinki, Finland) at 495 nm, and data were analyzed by Multiskan Ascent v2.6 software (Thermo Fisher Scientific SLU, Barcelona, Spain). The lower limits of detection were 1.95 ng/mL for sIgA and IgM.

IgA, IgM, IgG and IgG isotypes (IgG1, IgG2a, IgG2b, IgG2c) were quantified in plasma, milk, and SG and MLN homogenates by ProcartaPlex™ Multiplex immunoassay. Briefly, 96 well flat bottom plates were used to prepare samples following manufacturer’s instructions, as in previous studies (21). Data were acquired by MAGPIX^®^ analyzer (Luminex Corporation, Austin, TX, USA) at the Cytometry Service of the Scientific and Technological Centers of the University of Barcelona (CCiT-UB). The lower limits of detection were: 0.58 ng/mL for IgA, 1.70 ng/mL for IgG1, 1.73 ng/mL for IgG2a, 2.67 ng/mL for IgG2b, 3.67 ng/mL for IgG2c and 0.2 ng/mL for IgM. The relative abundance of IgG subtypes was analyzed considering total IgG. Thus, Th1 and Th2 responses were evaluated adding the levels of IgG subtypes, IgG2b + IgG2c and IgG1 + IgG2a, respectively.

### 2.8 Cell subset staining and flow cytometry analysis

Phenotypic population analysis was performed in MLN and splenic cells by flow cytometry analysis using fluorescent mouse anti-rat monoclonal antibodies (mAbs) conjugated to different fluorochromes. All the chosen mAbs were purchased from BD Biosciences (San Diego, CA, USA), Serotec (Kidlington, Oxford, UK) and Caltag (Burlingame, CA, USA): anti-TCR αβ (R73), anti-CD103 (OX-62), anti-NK (10/78), anti-CD62L (OX-85), anti-CD8α (OX-8), anti-CD4 (OX-35), anti-CD45RA (OX-33), anti-TCRγδ (V65). The staining combination and gates strategy is showed in **Supplementary Figure 1**. The staining technique was performed following the protocol previously described by Marín- Gallen et al. (22). Analyses were performed with a Gallios™ Cytometer (Beckman Coulter, Miami, FL, United States) in the CCiT-UB and data were analyzed by Flowjo v10 software (Tree Star, Inc., Ashland, OR, USA).

### 2.9 Cecal bacteria and Ig-coated bacterial analysis

The proportion of cecal bacteria and Ig-coated bacteria (Ig-CB) was determined as previously described (23) with slight modifications, only 10 µL of the homogenized cecal sample was used. A Cytek Aurora (Cytek Biosciences, Inc., CA, USA) flow cytometry equipment was used in the CCTi-UB. The acquisition parameters were adjusted to obtain a maximum of 25.000 counts. Data analysis was performed using the FlowJo v.10 software. The total bacterial and the Ig-CB proportions were evaluated as Massot et al. established before (24).

### 2.10 Gene expression analysis

SI and WAT samples kept in RNAlater were thawed and homogenized for RNA extraction and gene expression analysis. Samples were placed in lysing matrix tubes (MP biomedicals, Illkirch, France) and homogenized using a FastPrep-24 instrument (MP biomedicals, Illkirch, France). RNeasy Mini Kit (Qiagen, Madrid, Spain) was used for RNA extraction following the manufacturer’s instructions. RNA purity and concentration was determined with a NanoPhotometer (BioNova Scientific S.L., Fremont, CA, USA) and cDNA obtained using TaqMan Reverse Transcripiton Reagents (Applied Biosystems, AB, Weiterstadt, Germany). Then, Real Time (RT) – PCR for target genes (**Supplementary Table 1**) was performed with ABI Prism 7900 HT quantitative RT-PCR system (AB). Results were normalized using the housekeeping gene *Gusb* (β-glucuronidase, Rn00566655_m1, I) and analyzed using the 2-ΔΔCt method, as previously described (25). Data is shown as the percentage of expression in each experimental group normalized to the mean value obtained for the REF group, which was set at 100%.

### 2.11 Detection of *B. breve* M-16V

*B. breve* M-16V detection was carried out in fecal and mammary gland samples by qPCR technique following the protocol previously described by Gil-Campos et al. (26). Genomic DNA was extracted from ~ 100 mg of fecal or tissue samples using the FastDNA kit (MP biomedicals Inc., Santa Ana, CA, USA) following the manufacturer instructions. The probiotic genome was detected by Taq-Man based PCR assay. The forward, reverse and probe used were previously designed by Phavichitr et al. (27). The PCR was performed with ABI Prism 7900 HT quantitative RT-PCR system (AB) at the CCiT-UB services.

### 2.12 Microbial profiling of rat samples analysis

Total DNA was isolated from fecal (100 mg) and milk samples (500 µL-1 mL) using an automated assisted method based on magnetic beads (Maxwell^®^ RSC Instrument coupled with Maxwell RSC Pure Food GMO and authentication kit, Promega, Spain) following the manufacturer’s instructions with previous treatments to improve the DNA extraction. In brief, samples were treated with lysozyme (20 mg/mL) and mutanolysin (5 U/mL) for 60 min at 37°C and a preliminary step of cell disruption with 3-μm diameter glass beads during 1 min at 6 m/s by a bead beater FastPrep 24–5 G Homogenizer (MP Biomedicals). After the DNA extraction, DNA was purified using the DNA Purificaton Kit (Macherey-Nagel, Duren, Germany) following the recommended protocol and the final DNA concentration measured using Qubit^®^ 2.0 Fluorometer (Life Technology, Carlsbad, CA, USA). Microbial profiling was assessed by amplicon V3-V4 variable region of the 16S rRNA gene. Libraries were prepared following the 16S rDNA gene Metagenomic Sequencing Library Preparation Illumina protocol (Cod. 15044223 Rev. A). The libraries were then sequenced using 2x300 bp paired-end run on a MiSeq-Illumina platform (FISABIO sequencing service, Valencia, Spain). Negative and positive mock community (Zymobiomics) communities were also included.

Raw reads were then processed with the integrated dada2 method for denoising, amplicon sequence variance (ASV) clustering and chimeral removal. Reads were trimmed at 270 and 210 nucleotides in forward and reverse reads, respectively. Resulted ASV were then taxonomically assigned using Silva v.138. No rarefaction was done and also, samples with less than 4500 reads were removed and data was normalized using Centered-log-ratio (CLR). Beta diversity was based on Bray-Curtis distances and Permutational Analysis of Variance (PERMANOVA) was performed. Alpha-diversity indexes Chao1 and Shannon were also calculated and differences by group were assessed by Mann-Whitney and/or Kruskal-Wallis non-parametric test. Besides this, the Kruskall-Wallis test on the CLR normalized data were also assessed with Benjamini-Hochberg false discovery rate (FDR) correction. Taxa tables at phylum, family and genus level were provided to integrate to the other data obtained in the study. Negative binomial regression as implemented by DESeq2 tool was used for differential abundance analysis in order to estimate the fold-change of genus taxa (28). Plots were generated using MicrobeAnalyst platform v.2 (29).

### 2.13 Microbial metabolites profiling by SCFA analysis

SCFA analysis was performed using gas chromatography–mass spectrometry (GC-MS), following the method described by Eberhart et al. (30). An internal standard solution (3-Methylvaleric acid) was added to the samples that were processed and finally centrifuged at 4000 rpm for 2 min at 4°C according to the protocol. The final supernatant was collected, filtered-sterilized (0.22 μm PES size filter, Sarstedt SA) and then, injected in the Agilent GC 7890B–5977B GC-MS with a multipurpose sampler (Gerstel MPS, Mülheim, Germany). The GC column used was Agilent DB-FATWAX, 30 m × 0.25 mm × 0.25 μm, operated in split mode (20:1). The oven temperature program was set as follows: 100°C for 3 min, ramped to 100°C at a rate of 5°C min−1, then to 150°C for 1 min, further ramped to 200°C at a rate of 20°C min−1, and finally held at 200°C for 5 min. Helium was used as the carrier gas at a flow rate of 1 mL min−1, with an inlet temperature of 250°C. The injection volume was 2 μL. Standards curves for acetate, butyrate, and propionate were used for quantifying the SCFAs.

### 2.14 Statistical analysis

SPSS Statistics 22.0 software package (SPSS Inc., Chicago, IL, USA) was used for the statistical analysis. To assess normal distribution and homogeneity of variance of the data, Shapiro-Wilk and Levene test were used. Normal and homogeneous results were analyzed by one-way ANOVA. Kruskal-Wallis test was performed when results did not follow a normal and equal distribution to assess significant differences among groups (p<0.05). Spearman correlation coefficient was used to search correlation between variables. Non-metric multi- dimensional scaling (NMDS) in Rstudio using the R package vegan (31) was used to search clusters of similarities between samples in terms of immune factor composition. Besides, the function “envfit” assess the association of factors with the ordination of the samples in the NMDS. Differences were considered statistically significant when *p* value < 0.05.

## 3 Results

### 3.1 Animal body weight

**Figure 1:**
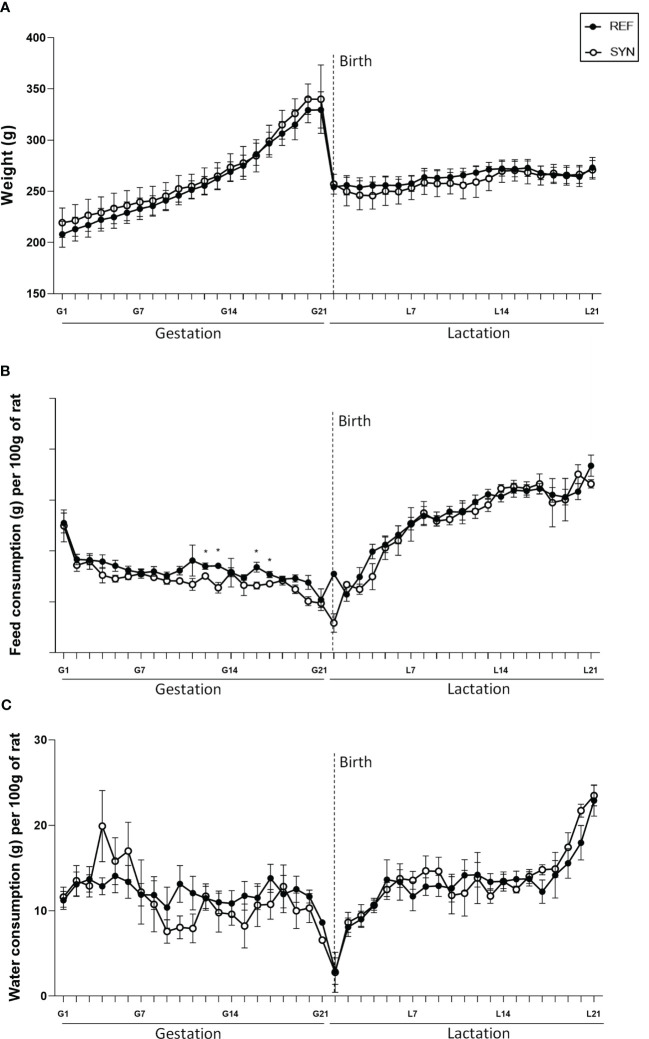
Effect of SYN supplementation on dams’ growth and daily intake during gestation and lactation. **(A)** Dams’ body weight (BW) evolution. **(B)** Dams’ chow intake. **(C)** Dams’ water intake. Data are expressed as mean ± standard error of the mean (S.E.M.). Statistical differences: ^*^ *p*< 0.05 *vs* REF. (n=5–6).

Synbiotic supplementation during gestation and lactation did not affect either the body weight gain or the overall daily food and water intake during pregnancy or lactation. Only punctual changes were observed in the food intake at the middle of the gestation (**Figure 1**). As expected, in all groups the body weight exponentially increased during gestation and showed a sharped decrease on the delivery day (**Figure 1A**). The overall intake showed a distinct pattern associated to the growth gain during gestation and the higher requirements of food and water during lactation (**Figures 1B, C**).

### 3.2 Organ size and growth parameters

At the weaning day, dams were measured and weighted, and different organs were obtained and weighted (**Supplementary Table 2**). No changes were observed due to the synbiotic supplementation in the body size evaluation. However, an increase in the relative weight of the SI was observed in the SYN dams.

### 3.3 Adipose tissue

**Figure 2:**
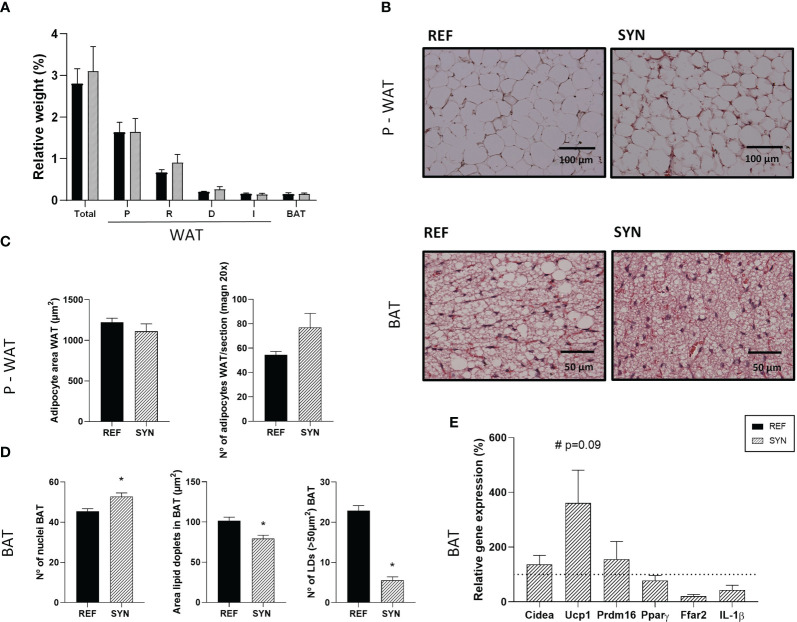
Impact of SYN supplementation on the adipose tissue. **(A)** Relative weight of the adipose tissue from different locations. **(B)** Hematoxylin and eosin-stained sections of parametric WAT (P-WAT) and BAT. Images were captured at 200x and 400x magnification, respectively. **(C)** Analysis of P-WAT: adipocyte area and number of adipocytes. **(D)** Analysis of BAT: number of nuclei, area of lipid droplets (LD), and number of LD (>50µm^2^). **(E)** Relative gene expression analysis of BAT calculated with respect to REF, which corresponded to 100% of gene expression (represented with a horizontal dotted line). Data **(A, C, D)** are expressed as mean ± S.E.M. Statistical differences: ^*^ *p*< 0.05 *vs* REF. (n=5–6). WAT, white adipose tissue; BAT, brown adipose tissue; P, parametric; R, retroperitoneal; D, dorsal; I, Inguinal.

The SYN administration during gestation and lactation provoked some changes in the adiposity of the rats (**Figure 2**). The nutritional intervention did not modify the relative weight of the adipose tissue from different body locations (**Figure 2A**). Representative images of histologic sections of WAT and BAT are shown in **Figure 2B** and **Supplementary Figure 2**. The number of adipocytes and the adipocyte area of the parametric-WAT were not modified with the supplementation (**Figure 2C**). In contrast, the BAT of the SYN group showed an increase in the number of the nuclei associated with a reduction of the area of the lipid droplets (LD) and the number of LD bigger than 50 µm^2^ (**Figure 2D**). Additionally, synbiotic supplementation showed a tendency to increase the relative gene expression of the *Ucp-1* gene (*p*=0.09) in WAT without affecting *Cidea*, *Prdm16*, *Pparγ*, free fatty acid receptor (*Ffar2)* and IL-1β gene expression (**Figure 2E**), suggesting a higher thermogenesis of the BAT.

### 3.4 Hematological variables

The last day of suckling, different hematological variables were analyzed in maternal blood samples (**Supplementary Table 3**). Synbiotic supplementation during gestation and lactation showed no effect on hematological parameters.

### 3.5 Fecal sample analysis

**Figure 3:**
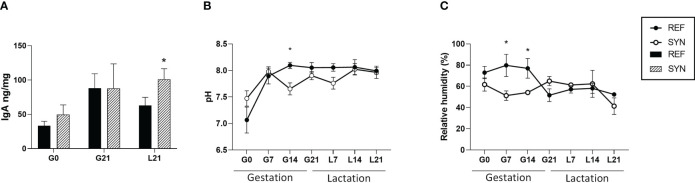
Fecal sample analysis throughout gestation and lactation. **(A)** IgA levels. **(B)** pH. **(C)** Relative water content. Data are expressed as mean ± S.E.M. Statistical differences: ^*^ *p*< 0.05 *vs* REF. (n=5–6). G, gestation; L, lactation.

Feces from the SYN group showed an increase in the total IgA at the end of the lactation (**Figure 3A**). Regarding to the pH and water content, feces SYN dams had overall lower pH and water content (during pregnancy) compared to REF (**Figures 3B, C**).

### 3.6 Immunoglobulin quantification

**Figure 4:**
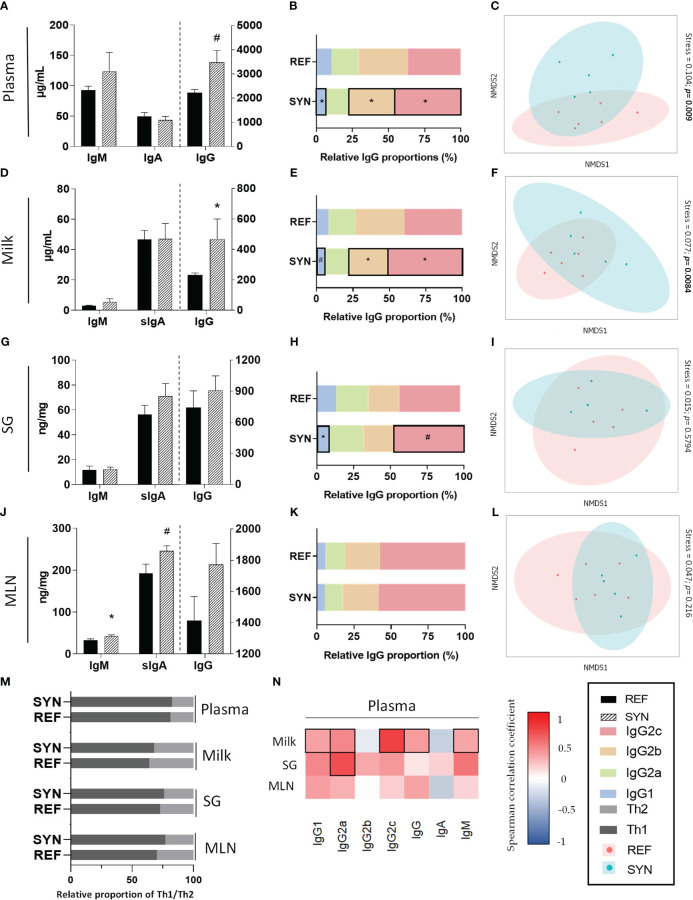
Effect of SYN supplementation on the Ig profiles in different compartments. Total Igs levels (IgA, IgM, and IgG) in **(A)** plasma, **(D)** milk, **(G)** SG and **(J)** MLN. Relative proportion of IgG subtypes in **(B)** plasma, **(E)** milk, **(H)** SG and **(K)** MLN. Analysis of non-parametric multidimensional scaling (NMDS) for the Ig profiles based on the Bray-Curtis distance in **(C)** plasma, **(F)** milk, **(I)** SG and **(L)** MLN. **(M)** Analysis of the Th1/Th2 ratio at the end of suckling in the different compartments. **(N)** Correlation between the Ig profile of plasma with respect to the Ig profiles of milk, SG and MLN. Data **(A, D, G, J)** are expressed as mean ± S.E.M. ^*^ *p*< 0.05 *vs* REF (by Kruskal Wallis test). (n=5–6). Each point represents an animal in figures **(C, F, I, L)** (n=11) (by ANOSIM test). The Spearman correlation coefficient is represented in the heat map following the color in the legend. Correlations with statistical significance (*p*< 0.05) are shown in a bold frame. SG, salivary gland; MLN, mesenteric lymph nodes.

To evaluate the impact of *B. breve* M-16V and scGOS/lcFOS during gestation and lactation on Ig levels, IgM, IgA and IgG concentration was determined in plasma, milk, salivary gland and MLN (**Figure 4**). Both in plasma (**Figure 4A**) and milk (**Figure 4D**) the total levels of IgG were increased due to supplementation without changes in IgA or IgM. Conversely, in MLN IgM was higher and IgA tend to be increased without changes in IgG (**Figure 4J**). The synbiotic supplementation did not influence the Ig levels in the salivary gland (**Figure 4G**). The relative proportion of IgG subtypes (IgG1, Ig2a, Ig2b, IgG2c) was also evaluated. Synbiotic supplementation modified the IgG subtype profiles in plasma, milk and SG (**Figures 4B, E, H**, respectively), mainly by increasing the relative proportion of IgG2c without changing the relative proportion of Th1/Th2associated response (**Figure 4M**). Moreover, non-metric multi-dimensional scaling (NMDS) graphs of each compartment were plotted with the Igs data and, in plasma (**Figure 4C**) and milk (**Figure 4F**) samples, different clusters appeared (p<0.01 with the ANOSIM test). Furthermore, plasma levels of IgG2c and IgG2b were highly correlated with the corresponding Ig in milk and in SG, respectively (**Figure 4N**). Additionally, the sIgA and IgM levels were quantified in the intestinal compartment in GW. Synbiotic supplementation did not affect either the sIgA (REF: 36.56 ± 6.72 μg/g; SYN: 28.98 ± 2.69 μg/g) or the IgM (REF: 0.14 ± 0.02 μg/g; SYN 0.09 ± 0.02 μg/g).

### 3.7 Phenotypic characterization of MLN and spleen cells

The relative proportion of MLN and spleen cell subsets was analyzed at the end of the study (**Table 1**). Spleen and MLN lymphocyte populations are rich in T cells with a low proportion of B lymphocytes. In terms of T helper (Th) and T cytotoxic (Tc) cells, Th predominates in both compartments. Regarding the minor populations of NK and NKT cells, the NK subset dominates in the spleen while the NKT subset prevails in the MLN. Synbiotic supplementation did not alter the proportion of B or T lymphocytes either in the spleen or in MLN. Additionally, the expression of CD8 was analyzed in the different subsets of lymphocytes, and only a punctual reduction in the proportion of Tc CD8+ cells occurred in MLN due to the supplementation. Additionally, adhesion molecules important in the intestinal homing such as αE integrin and CD62L were also assessed (**Supplementary Figure 3**). As expected, in both tissues CD62L was highly expressed and αE integrin was very low expressed. The synbiotic intervention did not modify the pattern expression of any of these molecules in the spleen or in MLN.

**Table 1:** Effect of *B. breve* M-16V and scGOS/lcFOS on the spleen and MLN immune cells proportion at the end of suckling period.

	SPLEEN		MLN	
%	REF	SYN	REF	SYN
**B cells (CD45RA+)**	9.13 ± 1.89	9.91 ± 4.09	13.64 ± 5.27	4.44 ± 5.71
**T cells (TCRαβ+NK- and TCRgδ+)**	72.23 ± 7.38	64.08 ± 8.67	79.90 ± 2.80	70.44 ± 17.04
TCRαβ+ NK-	64.87 ± 8.33	60.40 ± 10.76	77.83 ± 3.27	68.80 ± 17.35
% CD8+	27.83 ± 1.83	27.06 ± 1.52	22.63 ± 1.87	16.51 ± 4.31
TCRgδ+	2.09 ± 0.55	3.68 ± 2.11	2.61 ± 0.69	1.64 ± 0.32
% CD8+	2.56 ± 0.84	1.54 ± 0.35	3.42 ± 2.28	0.72 ± 1.43
CD4+ CD8-	49.15 ± 7.39	41.83 ± 9.31	61.88 ± 2.64	56.11 ± 7.32
CD8+ CD4-	24.72 ± 2.80	24.28 ± 3.68	20.28 ± 0.34	18.4 ± 0.72*
CD4+ CD8+	2.76 ± 0.59	1.45 ± 0.45	1.33 ± 0.26	1.00 ± 0.25
**NK (TCRαβ- NK+)**	4.45 ± 2.01	5.33 ± 2.73	0.85 ± 0.16	1.14 ± 0.54
% CD8+	55.86 ± 9.45	40.26 ± 18.52	21.47 ± 7.11	30.42 ± 11.90
**NKT (TCRαβ+ NK+)**	2.81 ± 0.85	3.77 ± 0.92	1.81 ± 0.44	1.80 ± 0.28
% CD8+	79.16 ± 5.46	86.30 ± 2.00	60.35 ± 7.37	64.80 ± 7.51
**αE+**	2.72 ± 0.40	4.47 ± 2.20	3.88 ± 0.76	8.04 ± 2.60
**CD62L+**	77.72 ± 0.33	72.81 ± 8.14	69.07 ± 10.40	52.90 ± 5.86

Data are expressed as mean percentage ± S.E.M. (n=5–6). Statistical differences: ^*^p< 0.05 vs REF. mAbs: fluorescein isothiocyanate (FITC), phycoerythrin (PE), peridinin chlorophyll protein (PerCP), allophycocyanin (APC), brilliant violet 421 (BV421), and phycoerythrin-Cyanine 7 (PE-Cγ7).

### 3.8 Gene expression analysis

**Figure 5:**
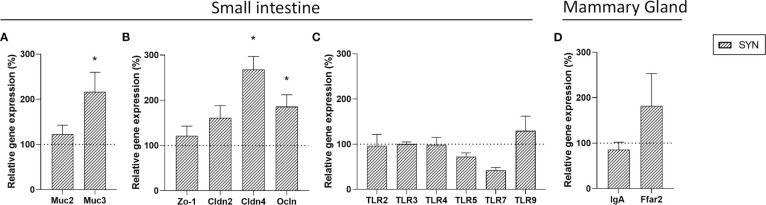
Gene expression in small intestine and mammary gland. Relative gene expression analysis in the small intestine of **(A)** mucins, **(B)** TJ proteins and **(C)** Toll-like receptors. **(D)** Relative gene expression of *IgA* and *GPR43* (*Ffar2*) genes in mammary gland. Relative gene expression was calculated with respect to REF, which corresponded to 100% of transcription (represented with a horizontal dotted line). Statistical differences: ^*^ *p*<0.05 *vs* REF. (n=5–6). *Muc2*, mucin2; *Muc3*, mucin3; *Zo*-*1*, Zonula occludens-1; *Cldn2*, claudin 2; *Cldn4*, claudin 4; *Ocln*, occludin; *TLR*, Toll-like receptor; *IgA*, immunoglobulin A; *Ffar2*, free fatty acid receptor 2.

The effect of *B. breve* M-16V and scGOS/lcFOS on gene expression in the intestine and mammary gland was assessed at the end of the lactation period (**Figure 5**). In the intestinal samples, genes implicated in the activity of the immune system and gut barrier were evaluated. Synbiotic supplementation increased the mRNA levels of *Muc3, Cldn4* and *Ocln*. No changes in TLR gene expression were observed in the intestine. Regarding the mammary gland, the SYN supplementation did not modify the expression of *IgA* or *Ffar2*.

### 3.9 IgA and Ig-CB cecal analysis

**Figure 6:**
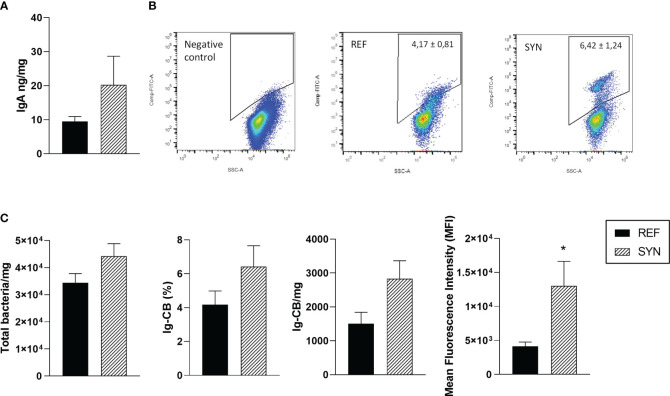
Evaluation of the cecal samples. **(A)** IgA concentration in cecum. **(B)** Representative dot-plots of Ig-CB. **(C)** Total bacterial counts in the cecum, proportion of Ig-CB, total Ig-CB and the mean fluorescence intensity (MFI) of the Ig-CB. Data are expressed as mean ± S.E.M. Statistical differences: ^*^ *p*< 0.05 *vs* REF. (n=5–6).

In the cecum, IgA is the most abundant Ig and can be bound to the resident bacteria (**Figure 6**). Synbiotic supplementation during gestation and lactation did not affect the IgA levels in the cecum (**Figure 6A**). The proportion of Ig-CB was evaluated in the cecum (**Figure 6B**), and after the supplementation, neither the total bacteria nor the relative proportion of Ig-coated bacteria were affected. However, the total Ig-CB tended to be increased (*p*=0.05). Furthermore, the amount of Ig coating of each bacteria was measured, by means of the Mean Fluorescence Intensity (MFI) of this population which was highly increased after the SYN supplementation (**Figure 6C**).

### 3.10 Intestinal histomorphometry

**Figure 7:**
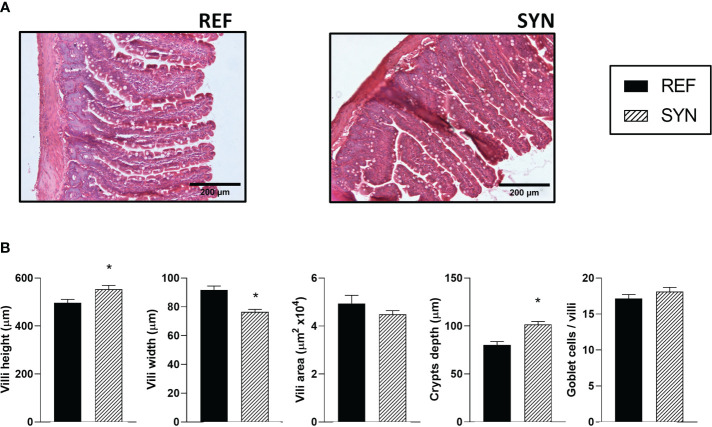
Effect of SYN on intestinal architecture. **(A)** Representative images of the small intestine stained with hematoxylin and eosin, 100X. **(B)** Height, width, and area of the intestinal villi. Results are expressed as mean ± S.E.M. Statistical differences: ^*^ *p*< 0.05 *vs* REF. (n=5–6).

The impact of the synbiotic on the intestinal architecture showed that the synbiotic supplementation during gestation and lactation induced histological changes in the intestinal epithelium (**Figure 7**; **Supplementary Figure 4**). Specifically, the villi height and the crypts depth were increased in the SYN group. On the contrary, the villi width was lower in the supplemented animals (**Figure 7B**). The synbiotic did not change the number of goblet cells responsible for mucus secretion.

### 3.11 *B. breve* M-16V detection in feces and mammary gland

**Figure 8:**
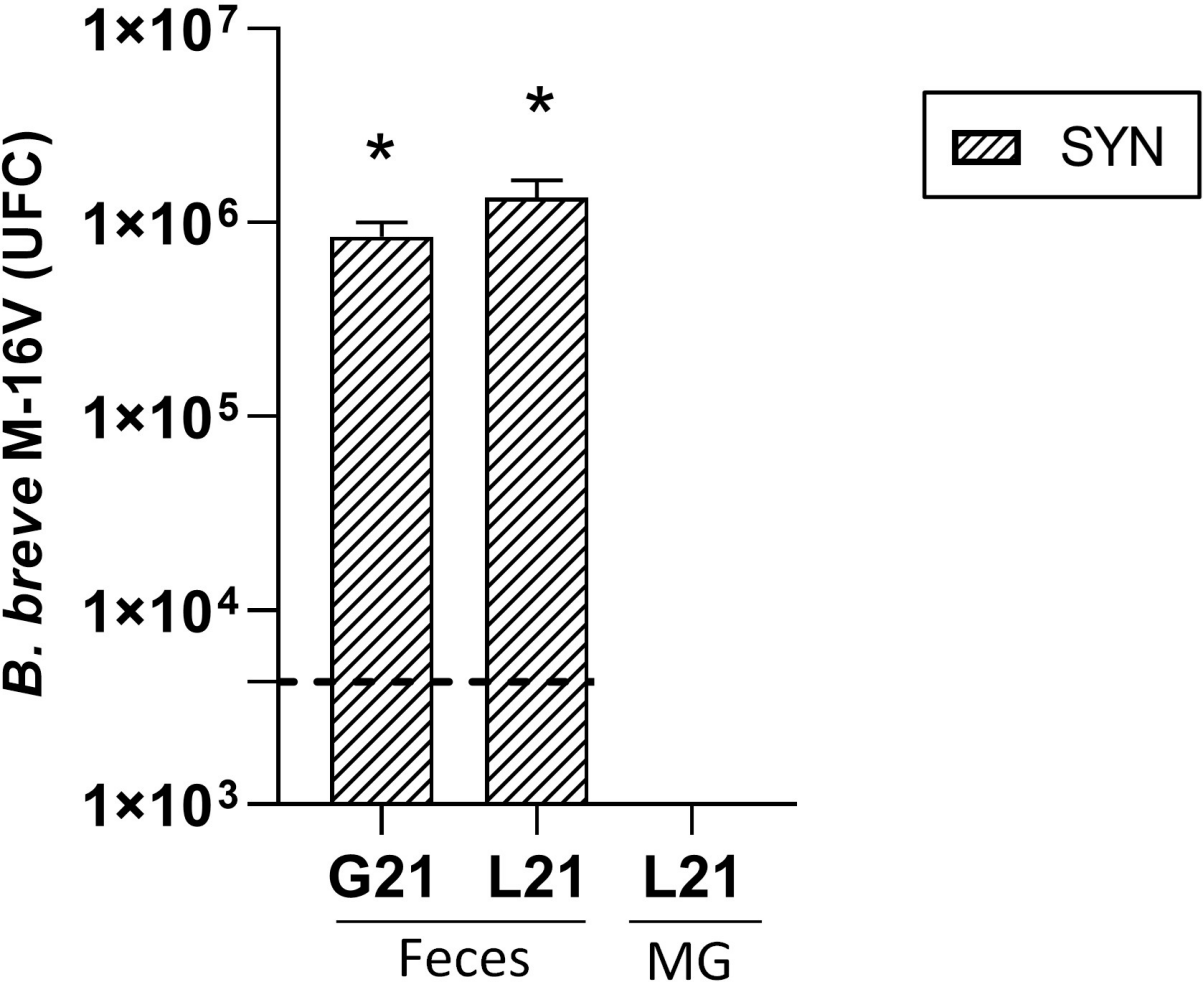
Detection of *B. breve* M-16V in fecal samples during gestation (G21) and lactation (L21) and in mammary gland (MG). The horizontal dotted line represents the levels of the REF group, which were considered as basal levels. Results are expressed as mean ± S.E.M. ^*^ *p*< 0.05 *vs* basal levels (n=5–6).

The presence of *B. breve* M-16V in feces and MG was studied also (**Figure 8**). In feces, both at the end of the gestation and lactation periods the number of *B. breve* M-16V UFC was increased 1000xin the supplemented dams with respect to the basal levels at the beginning of the intervention represented with the horizontal dotted line. The detection of *B. breve* M-16V in REF animals during gestation and lactation was always at basal levels (˜10EXP3). In order to explore the gut-mammary pathway its presence was also evaluated in MG. However, *B. breve* M-16V was not detected either in the SYN or the REF MG.

### 3.12 Impact of SYN supplementation on the rat intestinal and milk microbiota

Significant differences between groups and sample types were found (CC, CI and milk samples) as reported in the beta-diversity Bray-Curtis PERMANOVA and also, alpha-diversity indexes as well as taxonomical composition (**Supplementary Figure 4**).

**Figure 9:**
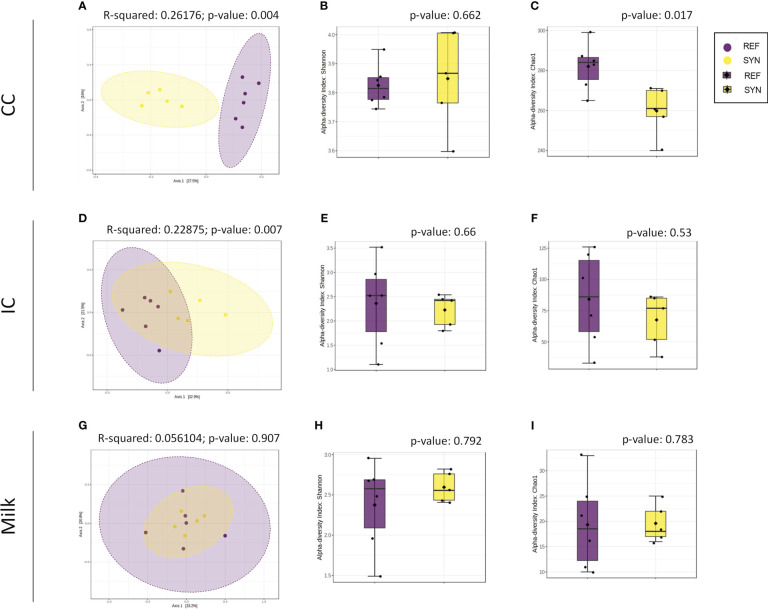
Beta- and alpha-diversity in rat microbiota depending on SYN intervention. Beta-diversity analysis using Bray-Curtis distance in CC **(A)**, IC **(D)** and milk samples **(G)**. Alpha-diversity indexes (Shannon index) and richness (Chao1 index) for CC **(B, C)**, IC **(E, F)** and milk **(H, I)** samples. Statistical testing was performed by PERMANOVA using Bray Curtis distances and the Mann-Whitney test was used for alpha-diversity indexes. (n=5–6).

SYN supplementation had an impact on the CC microbiota profile. Statistically significant differences were detected in the microbiota profiles of REF and SYN (permutational multivariate analysis of variance (PERMANOVA) *Bray-Curtis F-value*=3.1912; *R-squared*=0.26176; *p*=0.004) (**Figure 9A**), as well in the alpha-diversity indexes as measured by the Chao1 (*p*=0.01) and Shannon indices (*p*=0.662), respectively, (**Figures 9B, C**).

**Figure 10:**
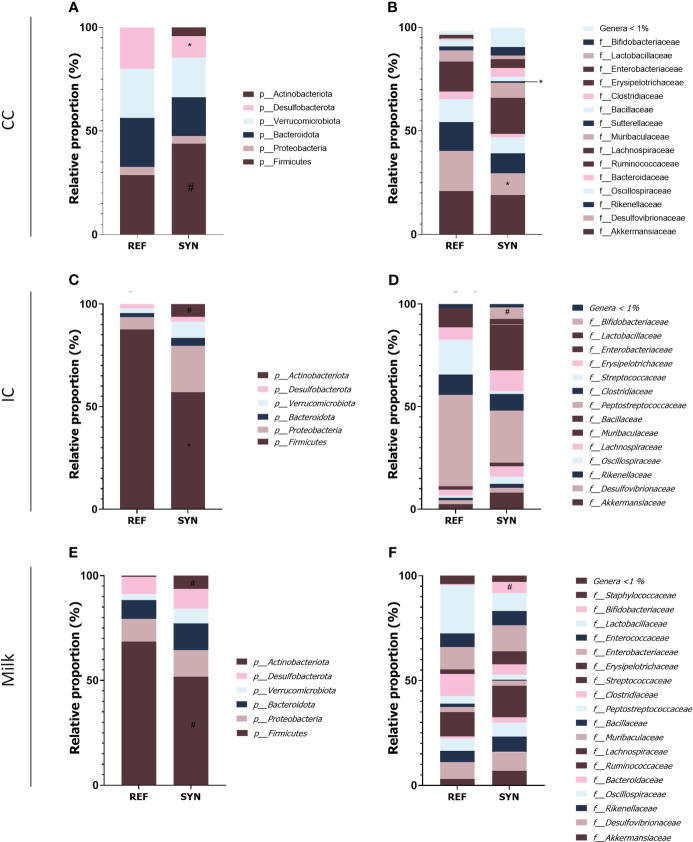
Microbiota composition of dams at weaning in cecal content (CC), intestinal content **(IC)** and milk. Relative proportions **(A)** phylum and **(B)** families present in the CC. Relative proportions **(C)** phylum and **(D)** families present in the IC. Relative proportions **(E)** phylum and **(F)** families present in the milk. Results are expressed as relative proportions of population. Statistical differences: ^*^ *p*< 0.05 *vs* REF; ^#^ *p*< 0.1 *vs* REF. (n=5–6).

Regarding the taxonomy of the CC, differences were found in the phylum, family and genera. In the SYN group, the proportion of *Firmicutes* tend (p=0.06) to be reduced while the abundance of *Desulfobacteria* was significantly reduced. The family analysis revealed that *Desulfovibrionaceae* and the *Suterellaceae* families were reduced in the SYN group (**Figure 10**). Thus, specific microbial genera were significantly present in SYN group including *Bifidobacterium*, *Faecalibaculum* (*Erysipelotrichidae* family) and *Marvinbruantia* (*Lachnospiraceae* family) and other butyrate producers such as *Blautia*, *Ruminoclostridium* and also, other *Lachnospiraceae* (**Table 2**).

**Table 2:** Differential microbial genera between REF and SYN in CC.

Genera	log2FC	lfcSE	p-values	FDR
**ɡ__*Anaeroplasma***	-23.783	2.4324	0.000	0.000
**ɡ*__Bifidobacterium***	8.6122	1.2076	0.000	0.000
**ɡ*__Faecalibaculum***	9.2997	1.3103	0.000	0.000
**ɡ*__Marvinbryantia***	8.8595	1.3216	0.000	0.000
**ɡ*__Eubacterium_ruminantium_group***	-8.72	1.3289	0.000	0.000
**ɡ*__Colidextribacter***	-1.2643	0.28954	0.000	0.000
**ɡ*__otherMuribaculaceae***	0.98846	0.33762	0.003	0.037
**ɡ*__Harryflintia***	1.2993	0.45857	0.005	0.044
**ɡ*__Ruminiclostridium***	1.627	0.61146	0.008	0.066
**ɡ*__Incertae_Sedis***	1.5033	0.57876	0.009	0.066
**ɡ*__Blautia***	4.9847	1.9217	0.009	0.066
**ɡ*__Lachnospiraceae_NK4A136_group***	1.2904	0.54075	0.017	0.108

DESeq2 results showing the log2 fold-change values of bacteria at genus level between REF and SYN groups (positive means more represented in SYN vs REF, and negative values means more represented in REF than in SYN).

The impact of the SYN supplementation was also observed in the intestinal content (IC) as two distant groups were identified. The beta-diversity analysis with Bray-Curtis distances reported significant differences (PERMANOVA] F-value: 2.6694; R-squared: 0.22875; p-value: 0.007) while no significant differences were observed in alpha-diversity indexes (**Figure 9**). The taxonomic analysis of the IC showed that the *Firmicutes* phylum was reduced and the *Actinobacteria* phylum tend to be higher in the IC of the SYN supplemented animals (*p*=0.07). With regard to the family diversity, the *Bifidobacteriaceae* tend to be increased in the SYN group (*p*=0.07) (**Figure 10**). The specific genera were overrepresented in SYN compared to REF group, including again the presence of *Bifidobacterium*, *Faecalibaculum* and *Blautia* (**Table 3**).

**Table 3:** Differential microbial genera between REF and SYM in IC.

Genera	log2FC	lfcSE	p-values	FDR
**ɡ*__Faecalibaculum***	9.9933	1.7576	4.945E-7	1.3013E-8
**ɡ*__Bifidobacterium***	9.6098	1.8423	3.4689E-6	1.8257E-7
**ɡ*__Eubacterium_ruminantium_group***	-10.295	2.1721	2.7154E-5	2.1437E-6
**ɡ*__Blautia***	7.8482	2.284	0.0050207	5.8997E-4
**ɡ*__Colidextribacter***	-6.5453	1.922	0.0050207	6.6061E-4
**ɡ*__Lactococcus***	-4.3031	1.5419	0.033301	0.0052581
**ɡ*__Ruminococcus_gauvreauii_group***	3.8752	1.8731	0.20928	0.038552
**ɡ*__Bacteroides***	-4.3033	2.2519	0.23335	0.056004

DESeq2 results showing the log2 fold-change values of bacteria at genus level between REF and SYN groups (positive means more represented in SYN vs REF, and negative values means more represented in REF than in SYN).

Regarding the milk microbiota profile, the SYN supplementation had no effect on the microbiota profile. No differences were found in beta-diversity and alpha-diversity between SYN and REF groups (**Figures 9G–I**). In general, milk microbiota in rats is characterized by a higher presence of *Firmicutes*. Our results indicated that the SYN supplementation tend to reduce the proportion of *Firmicutes* and *Actinobacteria* phylums (*p*=0.084 and *p*=0.088, respectively). In the family analysis, the *Bifidobacteriaceae* tend to be higher (*p*=0.07) in the SYN group (**Figure 10**). Finally, the genus analysis confirmed that SYN group had higher levels of *Bifidobacterium* compared to those observed in the REF group (6% *vs*. 1%, respectively but p-value FDR>0.05).

### 3.13 Cecal SCFA content

**Figure 11:**
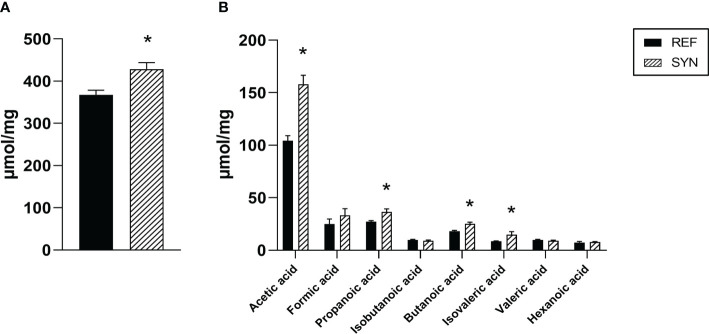
Cecal SCFA composition at the end of the weaning. **(A)** Total SCFA. **(B)** Acetic, formic, propionic, isobutanoic, butanoic, isovaleric, valeric and hexanoic acids were quantified by HS-GC-MS. Results are expressed as mean ± S.E.M. Statistical differences: ^*^ *p*< 0.05 *vs* REF. (n=5–6).

SCFA are the communication channel of the intestinal microbiota and the immune system. For this reason, the main SCFA were evaluated in CC at the end of the study (**Figure 11**). After the SYN supplementation, the total amount of SCFA was increased in the cecum (**Figure 11A**). This increase was mainly due to an increase in the total amount of acetic, propanoic, butanoic and isovaleric acids (**Figure 11B**).

### 3.14 Correlations between microbiota, SCFA and Igs

**Figure 12:**
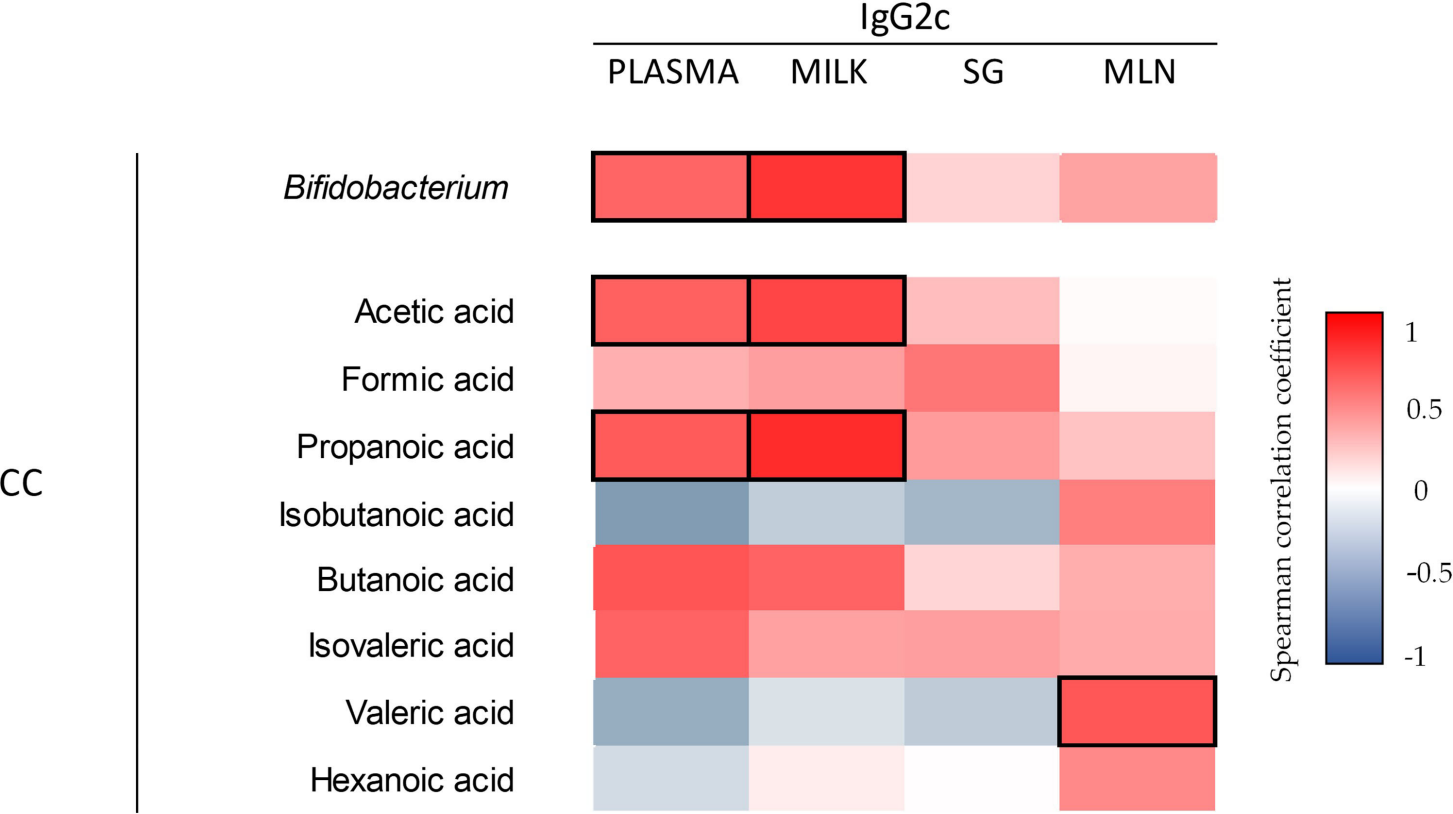
Correlation between the levels of Ig2c in plasma, milk, SG, and MLN with respect to the *Bifidobacterium* (16s sequence) and cecal SCFA. The Spearman correlation coefficient is represented in the heat map following the color in the legend. Correlations with statistical significance (*p*< 0.05) are shown in a bold frame. (n=5–6). SG; salivary gland; MLN, mesenteric lymph nodes; CC, cecal content.

Considering the importance of SCFA derived from the microbiota in the overall intestinal health, the correlation between SCFA-microbiota-Ig was evaluated (**Figure 12**). Acetic, propanoic, and butanoic acids were positively correlated with the cecal *Bifidobacterium* detected by sequencing the 16s gene (0.76, *p*=0.01; 0.79, *p*=0.006; 0.84, *p*=0.002, respectively). Likewise, acetic and propanoic acids were positively correlated with the cecal *Blautia* (0.73, *p*=0.01; 0.84, *p*=0.002, respectively). After the microbiota analysis, the correlation between the *Bifidobacterium* and the IgG2c of the plasma, milk, SG and MLN was performed. The cecal *Bifidobacterium* was positively correlated with the plasma and milk IgG2c. Furthermore, the correlation between the cecal SCFA levels and the concentration of IgG2c in plasma, milk, SG and MLN was assessed. Results suggest that plasmatic and milk IgG2c were positively correlated with the increased acetic and propanoic acids. Additionally, a positive correlation was observed between the IgG2c of the MLN with the valeric acid.

## 4 Discussion

During gestation and lactation, the maternal immune system undergoes significant changes to adapt to this critical period (32). To protect the fetus, a delicate balance between tolerance and defense is established, mediated by among others a unique Th1/Th2 response equilibrium. In early pregnancy, a shift towards a Th2 dominant immune response occurs, preventing fetal rejection. As gestation progresses, the immune response gradually becomes more balanced between Th1 and Th2, with Th1 primarily defending the fetus against pathogens. Maintaining this equilibrium is vital to ensure immune tolerance towards the fetus while allowing the maternal immune system to combat infections (33). After labor, breastfeeding further supports the infant’s immune balance by adjusting its composition based on the infant’s needs (34). This bridge between maternal and infant immune systems during gestation and lactation plays a vital role in safeguarding both mother and child’s health.

Additionally, maternal immunological status impacts the offspring’s development. In the last decades, probiotics, prebiotics and synbiotics have demonstrated beneficial effects in adult individuals (6). The research of synbiotics for immunological health improvement has been focused on prophylactic or complementary treatments for different diseases such as infections or antibiotics-induced diarrhea (9). However, few studies have evaluated the impact of synbiotic supplementation during gestation and lactation on the maternal and newborn immune system (35).

Here, we have demonstrated that the supplementation with a synbiotic composed of *B. breve* M-16V and scGOS/lcFOS during pregnancy is safe for the dams, since it does not affect the weight gain, food or water intake and the hematological parameters at the weaning day.

Physiologically, the expansion of adipose tissue during pregnancy aims to provide necessary nutrients for the correct development of the fetus (36). Dietary habits during pregnancy play an important role in the offspring health. Maternal high fat diet causes reprogramming of adipose tissue including increasing adipogenic and lipogenic markers in both WAT and BAT (37). The overexpansion of the adipose tissue during gestation may increase the adverse outcomes in the later life of both the mother and the offspring, mainly associated with glucose and insulin metabolism (38–40). In this study, supplementation with scGOS/lcFOS and *B. breve* M-16V during pregnancy, increased the number of nuclei and decreased the area of LDs in BAT. Moreover, the slight increase of *Ucp1* expression may be able to justify the obtained results pointing out the activation of this tissue (38, 41). BAT is specialized in dissipating excess energy into heat (non-shivering thermogenesis) through mitochondrial uncoupling protein 1 (*Ucp1*) (42). It is well documented that bioactive compounds like antioxidants and dietary fiber enhance the expression of thermogenic genes in BAT (43–45). In fact, BAT is involved in heat generation for maintaining the body temperature, and it is diminished in obese individuals. Thus, a higher number of nuclei and a reduction of the area of the LDs indicate a higher activation of the BAT (41). Joining these approaches, our results suggest that synbiotic supplementation influences positively the adipose tissue during gestation and lactation by increasing the BAT activity.

Different samples from the gastrointestinal tract were analyzed such as the SI, the cecum, and feces at weaning. *B. breve* M-16V and scGOS/lcFOS induced macroscopic and microscopic trophic effects on the SI. The relative weight of the SI and the villi and crypts length increased at the end of lactation, suggesting a higher nutrient-absorptive surface that could contribute to a healthier gastrointesinal tract and overall health (46). In addition to the observed morphologic changes resulting from supplementation, we also examined the immunological maternal status at systemic and gastrointestinal levels. The systemic immunological status was assessed by measuring the concentration of various Igs in different compartments, including plasma and milk. The supplementation of *B. breve* M-16V and scGOS/lcFOS enhanced the presence of IgG2c in both plasma and milk. In addition, a positive correlation between IgG2c levels in plasma and milk was found, suggesting the improvement of the immunological composition of milk. Human milk is known to contain high levels of IgA, which provides protection to the gastrointestinal tract. In contrast, rat milk primarily consists of IgG, which enhances short-term systemic immune response through receptor-mediated endocytosis in the neonatal intestine, facilitating its absorption (47). Similar to our results, previous studies have demonstrated that supplementation with the human milk oligosaccharide 2’-Fucosyllactose increases neonatal plasmatic IgG2c during early life (18) which also correlates in the maternal Ig profile of plasma and milk (48).

After infections IgGs are the main contributors to long-term immunity (49). Little is known about the role of dams IgG2c. However, in mice IgG3 (analog of rat IgG2c) (47) has been linked to regulatory responses in the neonate intestine to translocate microbes and might be involved in long-term immunity (50). Considering the increase of IgG2c in plasma and milk, we suggest that maternal synbiotic supplementation induces the IgG2c isotype switching to promote the long-term passive immunization to the infant through the breastfeeding.

The study of the gastrointestinal tract revealed that the synbiotic supplementation not only modified the microstructures of the SI but also the gene expression levels. The synbiotic supplementation increased *Muc3, Cldn4* and *Ocln* intestinal expression at the weaning day. *Muc3* is a transmembrane mucin that exerts protective roles in inflammatory bowel conditions (51). *Cldn4* and *Ocln* are epithelial tight junction (TJ) proteins involved in the passage of ions and macromolecules across the intestinal epithelium (52). The effects of the probiotic *B. breve* M-16V or the prebiotic scGOS/lcFOS have been widely studied by separate, suggesting anti-inflammatory and protective roles, respectively (53, 54). However, little information is known about the combination of both. Overall, these results indicate a direct relationship between the synbiotic supplementation and the improvement of the gut barrier function.

IgA is the most abundant Ig in the mucosal compartment and contributes to the development of the immune response (55). The analysis of Ig-CB has been controversial due to its imbalance in healthy and disease conditions (56). In heathy people, IgA-coated microbiome plays a homeostatic role in the gut favoring host-microbiome symbiosis while in inflamed gut it may exacerbate the inflammation (57). In our model in healthy conditions, the synbiotic supplementation, did not impact the sIgA, however it increased the Ig-CB in cecum, corroborating previous results that suggest the participation in the maintenance of the gut homeostasis (55, 58–61). The production of cecal IgA can be induced by endogenous or pathogenic bacteria, pathogen-induced IgA is considered to have high-affinity and specificity (62–64). This fact suggests that even though the IgA is not increased, the increased Ig-CB can be linked to the higher affinity induced by pathogenic species, facilitating its elimination.

Although there is limited information about the impact of synbiotics on cecal and fecal features during gestation and lactation, the effect of probiotic supplementation has been studied during these periods (10, 55). In our case, synbiotic supplementation reduced the fecal pH during gestation. Probiotics have been linked to a reduction of the intestinal pH due to the production of organic acids (61). As the cecum is characterized by the presence of high amounts of SCFA, and the synbiotic supplementation led to an overall increase in SCFA levels, primarily attributed to the rise in acetic, propanoic, butanoic, and isovaleric acids, both facts could be connected. In addition, numerous studies have shown that SCFAs have beneficial effects on energy metabolism, intestinal structure and integrity, as well as immunological regulation of anti-inflammatory activities (65). Overall, our study suggests that synbiotic supplementation enhance the production of SCFA which exert a reduction of the fecal pH.

Previously, it has been demonstrated that a maternal supplementation with a probiotic is able to influence the milk composition (66). The exact mechanisms involved are not yet fully understood, but both a direct and indirect effect could be participating. Regarding the direct effect of the probiotic, it could be due to its arrival to the breast milk by the entero-mammary pathway (67). This pathway enables the movement of commensal bacteria from the intestine to the mammary gland and milk, and consequently microorganisms are able to reach the infant intestine and therefore influencing the colonization of the newborn’s microbiota (66). This is not the case in our study, where we did not observe the presence of *B. breve* M-16V in the mammary gland.

Pregnancy leads to dramatic changes in the gut microbiome. In the last decades it has been demonstrated that during pregnancy a healthy microbiota undergoes a shift to a more dysbiotic one (68). In general, pregnancy reduces the alpha diversity and increases the beta diversity (69). Considering our results of the cecal microbiota, the synbiotic supplementation modified the beta diversity and also, the Chao1 diversity index and the cluster aggrupation of the supplemented group was different compared to the non-supplemented one. The SYN intervention also influenced the beta-diversity but not the alpha-diversity indexes in the IC. It is also relevant that SYN intervention did not influence the milk microbiota profile. However, the supplementation with the synbiotic influences the phylum, family and genus composition of the CC, IC and milk. In general, during gestation *Faecalibacterium* is reduced in the CC and IC (70). It has to be noted that *Faecalibacterium* is one of the main producers of butyrate (71). Our results indicated that the maternal synbiotic supplementation is able to counteract this reduction during this period, which can be confirmed with the levels of SCFA, as butyrate was increased in the SYN group. In addition, one of the most remarkable microbiota changes is the increase of the *Bifidobacteriaceae* and *Bifidobacterium* in the milk, suggesting that the maternal supplementation may be able to modify the milk composition through the entero-mammary route (67), as this change is also found in the CC. The importance of *Bifidobacterium* is due to the positive impact on the infant development, improving the nutrients absorption and regulating the immune system (72, 73). These findings confirm that this particular maternal synbiotic supplementation reduces microbiota alterations during gestation while enriches milk composition. These facts will contribute to the infant colonization and immune system maturation through the breastfeeding.

The obtained results point out that maternal nutritional intervention with *B. breve* M-16V and scGOS/lcFOS modulates maternal immune system during pregnancy and lactation. Synbiotic supplementation not only modulates the immunological profile of dams, but also the immune components of the milk composition which targets to encourage the infant development. Although the beneficial results of the study are clear, there are still some limitations and gaps that require further investigation. In this sense, although animal models have been highly useful in understanding the physiological changes induced after conception, there are significant differences between humans and animals, in this case rats, regarding pregnancy features. As consequence, integrating pre-clinical results into clinical knowledge should be performed to ensure translation. Besides this, to complete the study, further research is needed to determine whether supplementation during pregnancy or breastfeeding has a greater impact on modulating the maternal immune system and the composition of breast milk. Additionally, it is important to analyze the impact of *B. breve* M-16V and scGOS/lcFOS separately to evaluate if any of them interacts more with the maternal immune system. Finally, the last step required would be to analyze the impact of these maternal interventions on their offspring and study the transmission of the bioactive components from the mother to the infants.

## Data availability statement

The datasets presented in this study can be found in online repositories. The names of the repository/repositories and accession number(s) can be found in the article/**Supplementary Material**.

## Ethics statement

The animal study was approved by Ethics Committee for Animal Experimentation (CEEA) of the University of Barcelona (UB). The study was conducted in accordance with the local legislation and institutional requirements.

## Author contributions

LS: Data curation, Formal analysis, Investigation, Methodology, Writing – original draft. GK: Formal analysis, Investigation, Methodology, Writing – review & editing. BG: Formal Analysis, Investigation, Writing – review & editing. MM: Formal analysis, Investigation, Writing – review & editing. MB: Formal analysis, Investigation, Writing – review & editing. KK: Resources, Visualization, Writing – review & editing. JG: Resources, Visualization, Writing – review & editing. RB: Resources, Writing – review & editing. MC: Supervision, Writing – review & editing. MR: Conceptualization, Supervision, Writing – review & editing. MC: Conceptualization, Funding acquisition, Writing – review & editing. FP: Conceptualization, Funding acquisition, Supervision, Writing – review & editing.
