# Supplementary material for: Impact of maternal Bifidobacterium breve M-16V and scGOS/lcFOS supplementation during pregnancy and lactation on the maternal immune system and milk composition
Source: Front Immunol. 2024 Jun 21;15:1418594. doi: 10.3389/fimmu.2024.1418594 (PMC11224147; doi:10.3389/fimmu.2024.1418594)
Supplement: Supplementary file 1 [file DataSheet_1.pdf]

Supplementary Material

1 Supplementary Figures

1.1 Supplementary Figure 1

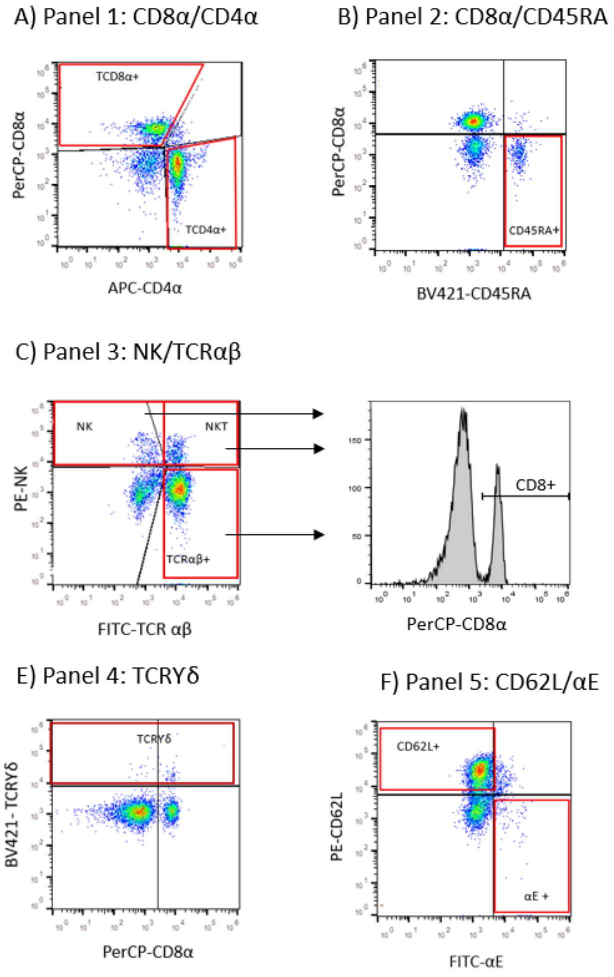

Supplementary Figure 1. Example of the gating strategy used for MLN and spleen lymphocytes.

Different panels were used to identify the different lymphocyte subsets in MLN and spleen samples. A) In panel 1, the different populations of T helper lymphocytes (TCD8α+ and TCD4α+ cells) were identified. B) In panel 2, the proportion of B cells (CD45RA+) were analyzed. C) In panel 3, NK and TCRαβ cells split, also the proportion of NKT cells was evaluated and in those, the proportion of CD8+ expressing cells. D) In panel 4, the relative proportion of TCRYδ was identified, including the TCD8a+TCRYδ+. E) In panel 5, the αE integrin/CD62L expression pattern was evaluated in populations from panel A and B.

1.2 Supplementary Figure 2

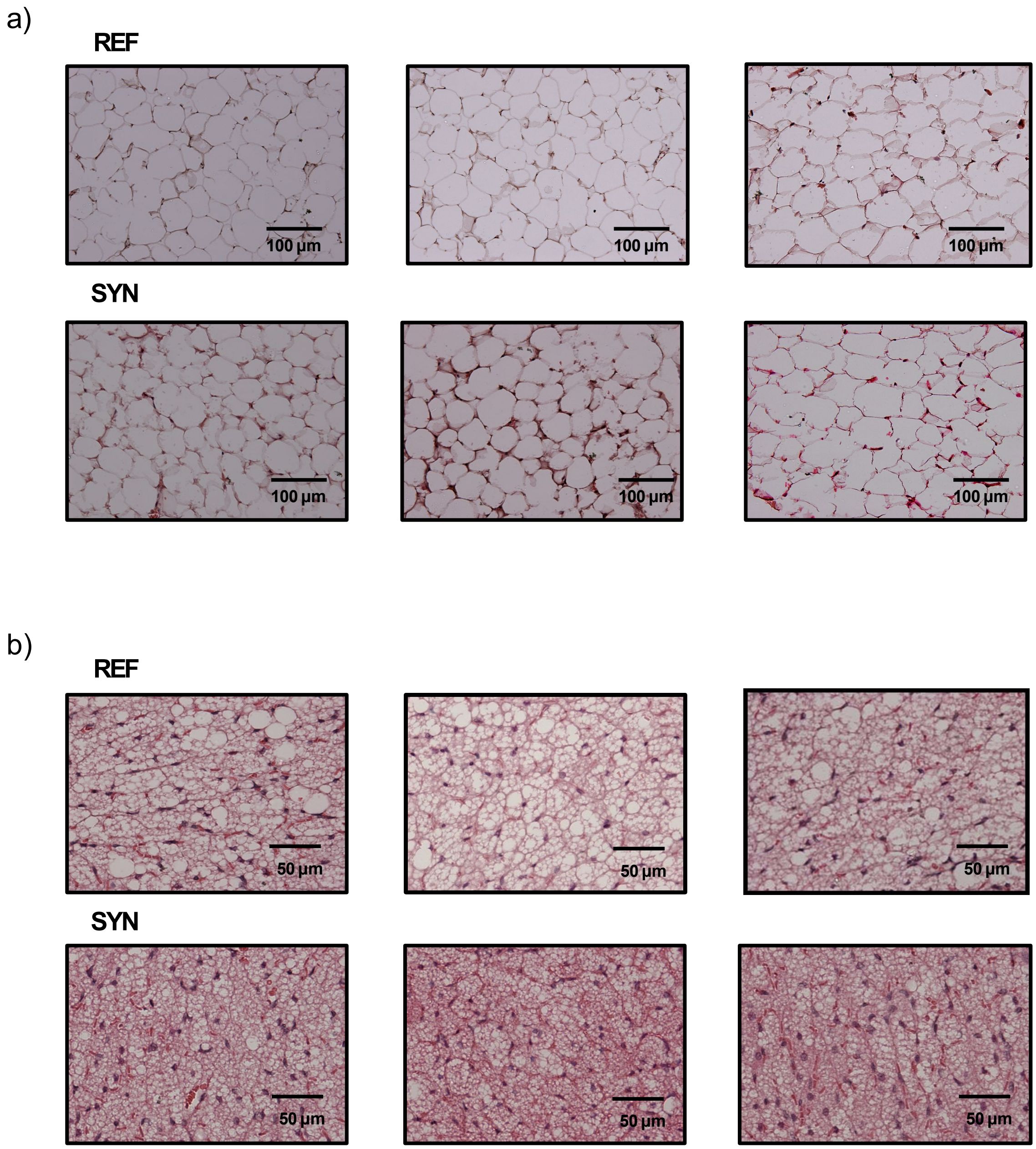

Supplementary Figure 2. Representative images of the (a) parametric adipose white adipose tissue (P-WAT) and (b) brown adipose tissue (BAT). Three representative images per group of hematoxylin and eosin-stained sections of WAT and BAT. Images were captured at 200x and 400x, respectively.

1.3 Supplementary Figure 3

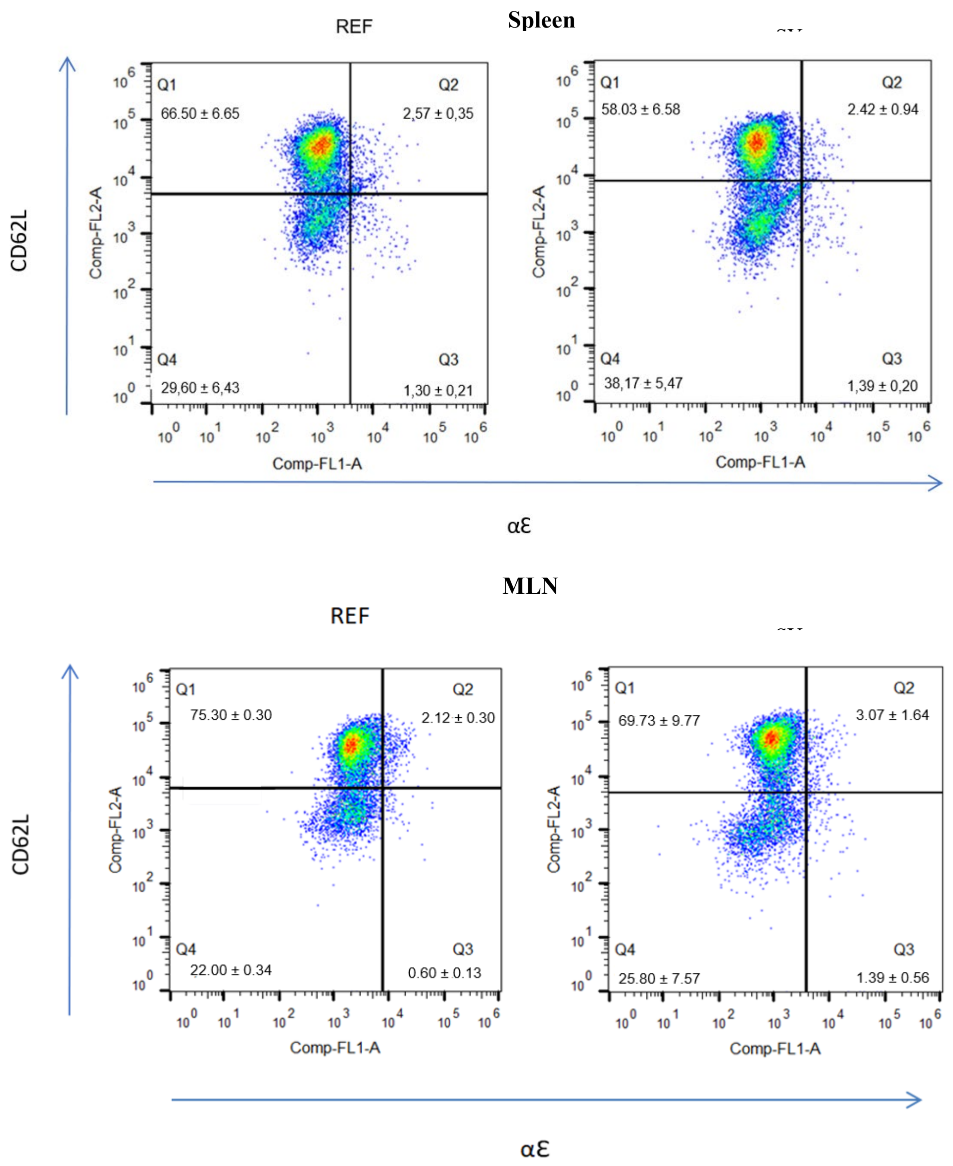

Supplementary Figure 3. Assessment of αE integrin/CD62L expression patterns in the spleen and MLN. Molecular pattern of αE integrin/CD62L in the total lymphocytes of the spleen and mesenteric lymph nodes (MLN) at the weaning day. Data are expressed as mean ± S.E.M. (n=5-6).

1.4 Supplementary Figure 4

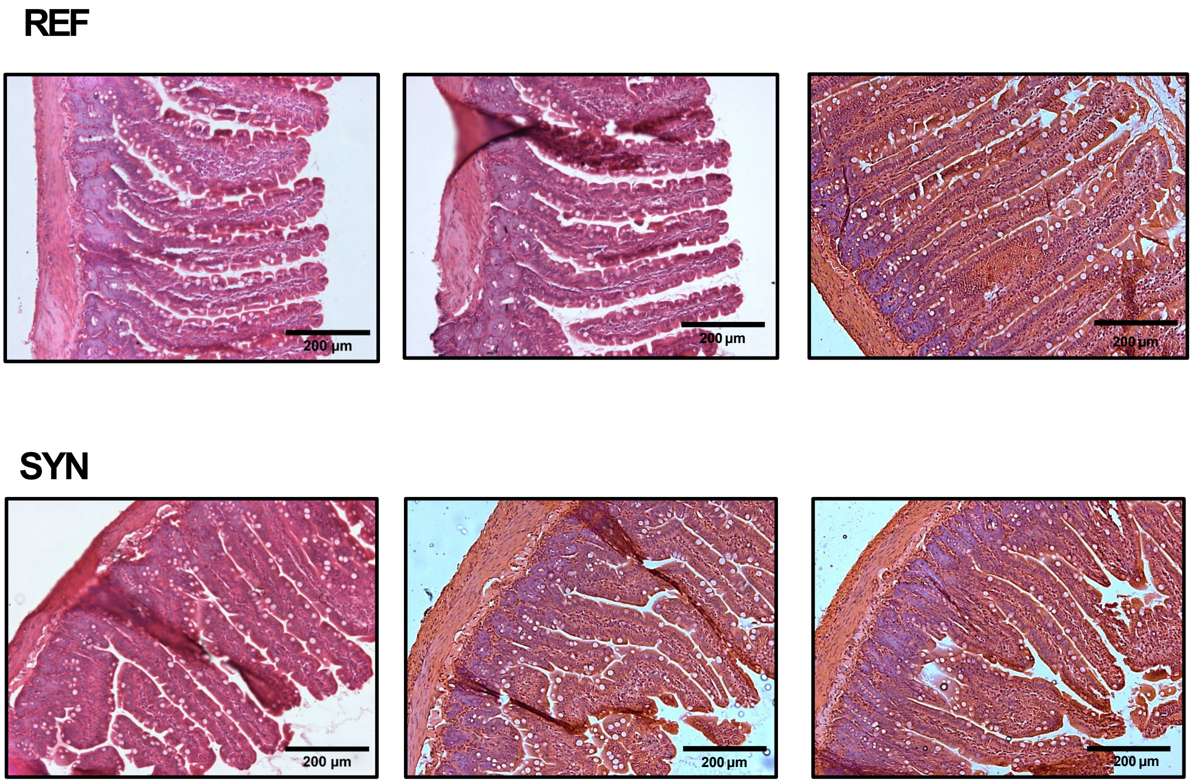

Supplementary Figure 4. Representative images of the intestinal structures. Three representative images per group of hematoxylin eosin-stained sections of the small intestine captured at 100x.

1.5 Supplementary Figure 5

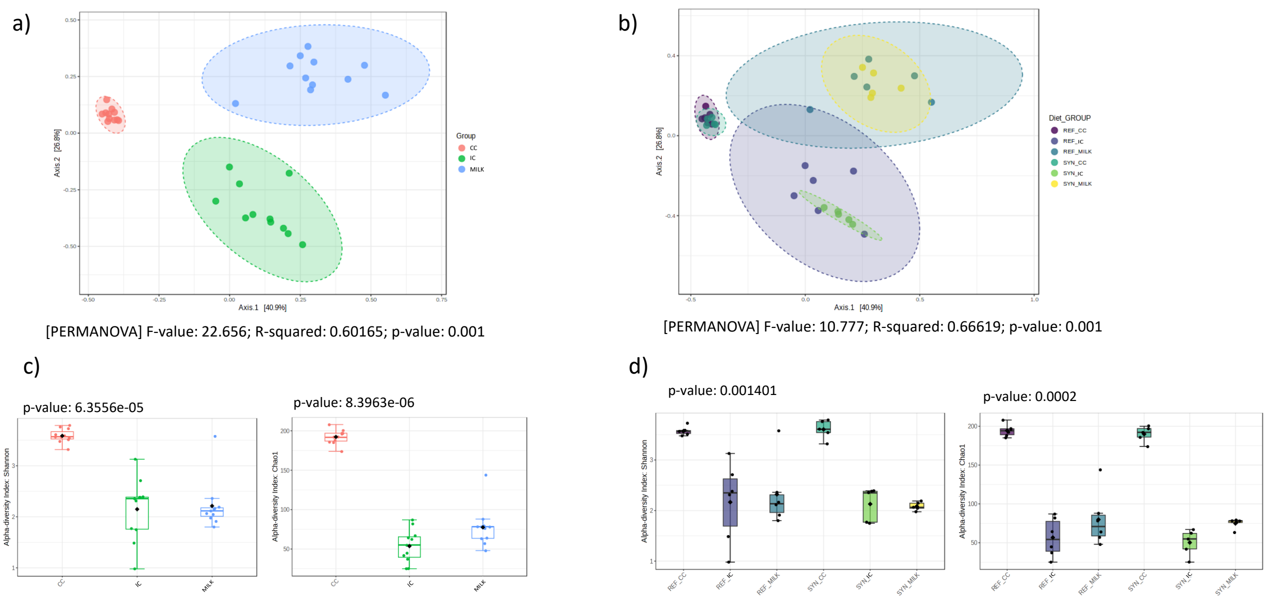

Supplementary Figure 5. Microbiota composition and diversity in cecal content (CC), intestinal content (IC) and milk samples from Beta-diversity analysis using Bray-Curtis distances according to (a) sample-type and also, (b) depending on the intervention group (REF *vs* SYN). Alpha-diversity indexes (Shannon index) and richness (Chao1 index) for according to (a) sample-type CC, IC and milk and (b) depending on the intervention group (REF *vs* SYN). Statistical testing was performed by PERMANOVA using Bray Curtis distances and the Mann-Whitney test was used for alpha-diversity indexes (n=5-6).

2 Supplementary Tables

2.1 Table 1. Description of the specific TaqMan primers AB.

Gene	Reference
Tlr2	Rn02133647_s1, I
Tlr3	Rn01488472_g1, I
Tlr4	Rn00569848_m1, I
Tlr5	Rn04219239_s1, I
Tlr7	Rn01771083_s1, I
Tlr9	Rn01640054_m1, I
Muc2	Rn01498206_m1, I
Muc3	Rn01481134_m1, I
Ocln	Rn00580064_m1, I
Cldn2	Rn02063575_s1, I
Cldn4	Rn01196224_s1, I
ZO1	Rn02116071_s1, I
IgA	331943, made to order
Cidea	Rn04181355_m1, I
Ucp1	Rn00562126_m1, I
Prdm16	Rn01516224_m1, I
IL-1β	Rn00580432_m1, I
Pparγ	Rn00440945_m1, I
Ffar2	Rn02345824_s1, I
Fcgrt	Rn00566655_m1, I, encoding for FcRn
Gusb	β-glucuronidase, Rn00566655_m1, I

I, Inventoried

2.2 Supplementary Table 2. Organ size and growth associated parameters at weaning

		REF	SYN
Body size	**Body length (cm)**	21.43 ± 0.39	21.22 ± 0.34
	**Body/tail length ratio**	1.19 ± 0.02	1.16 ± 0.03
	**BMI (g/cm^2)**	0.58 ± 0.03	0.60 ± 0.01
	**Lee index ($g^{0.33}/cm \times 10^3$)**	300.64 ± 6.91	305.38 ± 3.39
Relative organ size (%)	**Spleen**	0.14 ± 0.03	0.12 ± 0.04
	**Thymus**	0.14 ± 0.01	0.14 ± 0.00
	**Kidney**	0.36 ± 0.01	0.37 ± 0.02
	**Heart**	0.33 ± 0.05	0.38 ± 0.02
	**Liver**	5.52 ± 0.12	5.67 ± 0.17
	**Salivary gland**	0.14 ± 0.05	0.08 ± 0.00
	**Stomach**	0.57 ± 0.03	0.56 ± 0.02
	**Cecum**	0.32 ± 0.04	0.30 ± 0.05
	**Small intestine**	3.54 ± 0.05	4.50 ± 0.07*

Data are expressed as mean ± S.E.M. (n=5-6). Statistical differences: $^{*}p < 0.05$ *vs* REF.

2.3 Supplementary Table 3. Hematological variables at weaning day.

Hematological variables	REF	SYN
**Leucocytes (x10^9 /L)**	7.83 ± 1.07	6.95 ± 1.29
**Lymphocytes (x10^9 /L)**	2.30 ± 0.41	2.78 ± 0.19
**Monocytes (x10^9 /L)**	0.42 ± 0.06	0.30 ± 0.04
**Granulocytes (x10^9 /L)**	4.58 ± 0.89	3.88 ± 1.30
**Erythrocytes (x10^{12} /L)**	9.28 ± 0.51	9.53 ± 0.37
**HGB (g/L)**	162.17 ± 8.29	168.75 ± 7.48
**HCT (%)**	47.82 ± 2.72	49.40 ± 1.83
**MCV (fL)**	51.57 ± 0.30	51.90 ± 0.33
**MCH (pg)**	17.47 ± 0.27	17.68 ± 0.31
**Platelets (x10^9 /L)**	177.83 ± 47.20	232.25 ± 25.32

Data are expressed as mean ± S.E.M. (n=5-6). HGB, hemoglobin; HCT, hematocrit; MCV, mean corpuscular volume; MCH, mean corpuscular hemoglobin.
